# Neuromechanical interference of posture on movement: evidence from Alexander technique teachers rising from a chair

**DOI:** 10.1152/jn.00617.2013

**Published:** 2014-05-14

**Authors:** Timothy W. Cacciatore, Omar S. Mian, Amy Peters, Brian L. Day

**Affiliations:** UCL Institute of Neurology, London, United Kingdom

**Keywords:** posture, movement, balance, muscle tone, sit-to-stand, Alexander technique

## Abstract

While Alexander technique (AT) teachers have been reported to stand up by shifting weight gradually as they incline the trunk forward, healthy untrained (HU) adults appear unable to rise in this way. This study examines the hypothesis that HU have difficulty rising smoothly, and that this difficulty relates to reported differences in postural stiffness between groups. A wide range of movement durations (1–8 s) and anteroposterior foot placements were studied under the instruction to rise at a uniform rate. Before seat-off (SO) there were clear and profound performance differences between groups, particularly for slower movements, that could not be explained by strength differences. For each movement duration, HU used approximately twice the forward center-of-mass (CoM) velocity and vertical feet-loading rate as AT. For slow movements, HU violated task instruction by abruptly speeding up and rapidly shifting weight just before SO. In contrast, AT shifted weight gradually while smoothly advancing the CoM, achieving a more anterior CoM at SO. A neuromechanical model revealed a mechanism whereby stiffness affects standing up by exacerbating a conflict between postural and balance constraints. Thus activating leg extensors to take body weight hinders forward CoM progression toward the feet. HU's abrupt weight shift can be explained by reliance on momentum to stretch stiff leg extensors. AT's smooth rises can be explained by heightened dynamic tone control that reduces leg extensor resistance and improves force transmission across the trunk. Our results suggest postural control shapes movement coordination through a dynamic “postural frame” that affects the resistive behavior of the body.

difficulty performing whole body movements can occur at all levels of ability. For instance, a skilled dancer might struggle to perform an arabesque, while an older individual might struggle to rise from a chair. These are very different tasks, but they have something fundamental in common. Any action involving the whole body requires solving simultaneously component motor tasks, namely *1*) executing the movement plan, *2*) keeping the body's mass balanced above the base of support, and *3*) preventing postural collapse against gravity (Hess 1943; Massion 1992). It is possible that action difficulties arise from the orchestration of these component tasks. This could happen when the solution to one component interferes with performance of another. In the case of rising from a chair, a possible manifestation of such interference is the “sit-back” failure sometimes experienced by healthy elderly people (Riley et al. 1997). Of course, these failures could simply stem from insufficient strength (Brown et al. 1995; Moxley Scarborough et al. 1999), flexibility (Fleckenstein et al. 1988; Gleim and McHugh 1997) or practice (Mouchnino et al. 1992). However, the sit-to-stand (STS) movement does not require substantial flexibility, and elderly adults certainly do not lack practice. While strength is a contributing factor (Corrigan and Bohannon 2001; Lord et al. 2002; Moxley Scarborough et al. 1999; Schenkman et al. 1996), it cannot account fully for their difficulty as those affected are generally strong enough to perform the task (Ploutz-Snyder et al. 2002; Schultz et al. 1992; Yoshioka et al. 2007). A clue comes from the observation that healthy elderly adults exhibit counterproductive movement features, such as leaving the chair with their body mass further backward and under-foot pressure further forward than young adults (Mourey et al. 2000; Schultz et al. 1992), both of which exacerbate performance difficulty. Paradoxical motor features such as these are compatible with motor interference effects. The STS action, therefore, might be a good paradigm for investigating mechanisms of interference between the component motor tasks of movement, balance and posture.

In theory, anti-gravity postural support has considerable potential to interfere with movement because it requires muscle activity that is spatially complex, dynamic and ever-present (Gurfinkel et al. 2006), which could act to obstruct motion. A prediction emerging from this hypothesis is that differences in postural control between individuals should lead to differences in the interference effect and consequently in performance of whole body actions such as STS. A relevant observation is that postural support, when active during stance, was found to have substantially different properties in a group of subjects trained to a high level in the Alexander technique (AT) compared with an untrained but otherwise healthy group (Cacciatore et al. 2011a). In essence, the AT group had lower, or more adaptive, hip-joint and axial postural stiffness, as revealed by reduced resistance to externally applied slow mechanical perturbations. Furthermore, in a separate study, the two groups were found to perform the STS action differently (Cacciatore et al. 2011b). When the participants were instructed to rise from a chair at a self-selected speed, but to perform the movement as smoothly as possible without using momentum, the two groups chose similar movement durations, but the AT group achieved a much smoother rise. These two studies, therefore, provide some initial support for the hypothesis that the neuromuscular system for posture interferes with movement. However, it is necessary to rule out other reasonable explanations. For example, the healthy subjects may simply have misunderstood the instructions or been given insufficient feedback about their movements to modify their STS performance appropriately. Another objection is that, because the STS movement was performed at a self-selected speed, participants may have used their habitual movement pattern, thus merely reflecting differences between a trained and an untrained habit. It is also possible that the force distribution between leg joints was somehow different between the two groups, allowing for a smoother execution by the AT group.

In the present study we explore these alternative explanations by measuring the whole body kinematics and kinetics in AT teachers and healthy untrained (HU) adults attempting to rise smoothly from a chair under strict temporal and postural constraints. We carefully controlled the position of the body and the feet with respect to the seat edge to minimize biomechanical differences between the groups, and joint moments were calculated to check for any differences in force distribution between joints. Set movement durations ranging from 1 to 8 s were targeted, with feedback of actual movement duration being given after each trial. The rationale is that the necessity to execute unnaturally slow movements (8-s duration) should eliminate any misunderstanding of the instruction to rise from the chair smoothly without using momentum, and reduce any tendency to resort to habitual movement patterns. If the previously observed performance difference between these two groups were due to such factors, we would expect the difference to get smaller, or even disappear, with increasingly slower movements. As an additional manipulation, the movements were performed with the feet in three standardized anteroposterior positions, making the task more difficult the further forward the feet. Again, we would expect the increase in task difficulty to affect the performance of the two groups equally for all movement speeds. Contrasting predictions arise from the alternative hypothesis, which is that jerky movement arises from interference between motor systems, making it mechanically necessary to generate momentum to rise from the chair. Then, the slower the movement and further forward the feet, the greater will be the need to inject extra momentum, hence the greater will be the difference in performance between the two groups.

## METHODS

### Ethical Approval

This study was approved by the UCL Research Ethics Committee and was in accordance with the Declaration of Helsinki. All subjects provided written, informed consent.

### Subjects

#### Healthy adults.

A total of 10 HU adult subjects aged 28–65 were studied (4 men, 6 women). Volunteers had no medical conditions affecting daily activities or history of lower limb surgery and could rise from a chair without difficulty or pain. HU had a mean age of 44.0 ± 12.0 yr, mass of 74.3 ± 10.4 kg and height of 171.5 ± 8.1 cm.

#### AT teachers.

Ten teachers who had completed a 1,600-h course certified by the Society of Teachers of the Alexander Technique participated in this study. AT teachers and HU adults were individually matched in sex and age (within 5 yr) and approximately matched in height and body mass. The mean age, height and weight of AT was 44.9 ± 10.7 yr, 69.9 ± 9.9 kg and 174.0 ± 10.7 cm, respectively.

### Experimental Protocol

#### Setup.

**Fig. 1.**
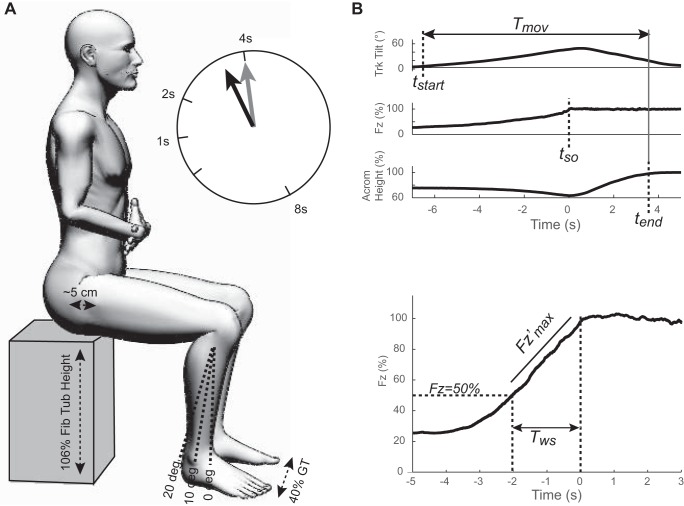
Experimental setup. *A*: subjects stood up from a standardized position with the chair at 106% of fibular tuberosity (Fib Tub) height, feet width at 40% of greater trochanter (GT) stance height and the GT positioned ∼5 cm from the front chair edge. Three anterior-posterior foot positions were used and given by 20°, 10° and 0° of shank orientation relative to vertical. Subjects aimed to perform the movement at a uniform speed that was directed to make the total movement last 1, 2, 4 or 8 s. Immediately following each trial, a biofeedback display indicated the actual (black arrow) vs. instructed (gray arrow) movement durations. To prevent interfering with the task, feedback was not given during the trial. *B*: quantification of movement time (*T*_mov_) and weight shift. Sit-to-stand (STS) *T*_mov_ was calculated from the onset of trunk (Trk) lean to when the acromion (Acrom) reached a vertical threshold. Weight shift was quantified by the maximal derivative ${\mathrm{(F}}_{z}^{\prime }$ max) and the duration of the final 50% of weight shift (*T*_ws_). F_*z*_, vertical force; *t*_start_, start time; *t*_SO_, seat-off time; *t*_end_, end time.

Participants were seated on a custom-built height-adjustable stool instrumented with a force plate seat (model 9286A, Kistler Instrumente, Winterthur, Switzerland) with each foot resting on a floor mounted force plate (model 9281B, left leg; model 9287, right leg). The bias of all force plates was reset following each trial to minimize drift. The chair was adjusted to 106% of the height from the floor to fibular tuberosity. Subjects sat with their greater trochanter ∼5 cm from the forward chair edge to minimize thigh contact and enable calculation of the hip moment prior to seat-off (SO). Three foot positions were used, with the shank at 20, 10 and 0° relative to vertical (Fig. 1). The feet were placed at width of 40% of floor to greater trochanter height.

#### Task.

Subjects were asked to stand up at four different speeds, with the total movement duration of 1, 2, 4 and 8 s (Fig. 1*A*). They performed two trials of each of the 12 conditions (4 speeds × 3 foot positions). To increase accuracy for performing these different movement durations, visual feedback was presented after the trial, depicting the instructed and actual movement times on a circular dial. Trial order was blocked by movement time but randomized across foot positions, so that posttrial feedback could be applied to the subsequent trial. This time-feedback was computed using an inertial sensor (XSENS, MTx) and LabVIEW (National Instruments, Austin, TX), as the duration from when the trunk inclined forward 10° at movement start (relative to the initial seated position) to when it again crossed this value after SO, during the extension phase. Subjects were asked to rise from the chair at a constant, uniform speed. Subjects clasped their hands together loosely in front of their trunk and were instructed not to use their arms to aid the movement. Prior to data collection, all participants practiced chair rises at each speed until they could accurately achieve the desired movement duration.

#### Data collection.

A CODA motion capture system (6 × Cx1 units, Charnwood dynamics, Rothley, UK) was used to capture, at 100 Hz, the three-dimensional (3D) positions of 52 infrared emitting diodes (IREDs) and kinetic data. IREDS were mounted to enable bilateral tracking of limb segments (both feet, shanks, thighs, upper arms, forearms) and axial segments (pelvis, 3 trunk segments, head). With the exception of the upper arms, rigid clusters of four noncollinear IREDs were securely affixed to each body segment.

### Kinetic Model

Raw kinematic and kinetic data were processed using Visual3D version 4 software (C-Motion, Germantown, MD) to obtain a 15-segment model of the body for each subject. In preexperiment calibration trials, a digitizing pointer was used to identify the location of various anatomical landmarks. Additionally, participants traced circles ∼30 cm in diameter with each foot and had their leg passively flexed and extended at the knee over a 40° range while seated to enable estimation of functional hip joint center and knee flexion-extension axes (Schwartz and Rozumalski 2005). The anatomical landmarks and functional joints were used to define segment fixed 3D local coordinate systems (LCS) tracked by the marker clusters (e.g., Cappozzo et al. 1995). The upper arm segments were defined and tracked using virtual points constructed at the shoulder and elbow as subject-specific offsets from the upper back and trunk LCS. Segments' mass, moments of inertia, and center of mass (CoM) were determined from regression models (Dempster 1955; Hanavan 1964) and used to compute whole body CoM position. Force plate data, segment kinematics, and segment inertial properties were used to perform 3D inverse dynamics for the computation of flexion-extension joint moments in segment coordinates about the mediolateral axis of the proximal segment. For computing offline movement duration a single combined trunk segment was defined from the acromions (subject-specific offsets from the upper back LCS) and hip joint centers (subject-specific offsets from the pelvis LCS).

### Data Analysis

Data analysis was performed in MATLAB (The Mathworks, Natick, MA) version R2009a. Quantities were computed for individual trials and averaged across repetitions within a subject.

#### Movement duration.

We quantified the total movement duration by computing an offline measure, *T*_mov_, that was more accurate than that used for posttrial feedback. *T*_mov_ was computed as the duration between when the combined trunk segment inclined 3° from the initial seated position to when the vertical mid-acromion height exceeded 98% of its maximal value (Fig. 1*B*).

#### Weight shift.

Weight shift was quantified by its rise time, the maximal rate of loading and maximal leg extensor moments (Fig. 1*B*). The weight-shift rise time, *T*_ws_, was computed between when the combined feet vertical force (F_*z*_) was last below 50% body weight (BW) until it first exceeded 98% BW, which was defined as SO. The last half of weight shift was chosen because it was highly stereotyped and reflected behavior related to the critical moment of lift-off. The maximal rate of combined-foot vertical loading, $F_{z}^{\prime }$ max, was calculated by low-pass filtering F_*z*_, (15 Hz, bidirectional 4th-order Butterworth) and taking the maximum of the derivative before SO. Maximal hip and knee extensor moments were calculated as the maximum of the bilateral averages for each joint normalized by a subject's BW.

#### CoM velocity.

Forward movement was quantified by CoM velocity in the anterior-posterior direction, *V*_CoM_, as calculated by Visual3D.

#### Bipedal balance.

Bipedal balance at lift-off was assessed by the anterior/posterior distance between the whole body CoM and combined-foot center of pressure (CoP) averaged over a 50-ms interval centered around SO as: *X*_CoM-CoP_ = CoM − CoP. Positive values indicate the CoM was anterior to the CoP at SO.

### Statistics

Statistical significance was determined using a three-way mixed design ANOVA (speed × foot position × group) using SPSS version 19.001. A Huynh-Felt correction was made for comparisons where Mauchly's Test indicated a violation of sphericity (at *P* < 0.05). We chose α < 0.01 for the significance level to minimize false positives from multiple comparisons. Correlations between variables were computed using linear correlation (Pearson's coefficient) and principal component analysis. All reported errors are SD, unless otherwise noted.

## RESULTS

Subjects were able to vary their *T*_mov_ to match approximately the instructed times (1.54 ± 0.30, 2.36 ± 0.29, 4.32 ± 0.58 and 8.15 ± 1.59 s, for 1-, 2-, 4- and 8-s conditions, respectively). This resulted in a main effect of speed on *T*_mov_ [speed: *F*(1.43,25.7) = 483.5, *P* < 0.001], but there were no significant effects of group, foot position or any interactions (*P* > 0.01). Despite this similarity, there were clear and profound differences in the performance of the two groups. These differences were apparent during the first half of the movement up to the point of SO and were most evident for the slower movements. [See Supplemental Material for videos of an 8-s chair rise from the midfoot position (HA subject, video 1; AT teacher, video 2); The online version of this article contains supplemental data.] Videos show the inverse 3D model from a single trial for each subject; blue arrows indicate feet and seat contact forces. In general, the AT group performed the movements smoothly with simultaneous feet-loading and forward flexion. In contrast, the movements of the HU group were jerkier with complex feet-loading patterns and a greater reliance on forward CoM momentum (rather than CoM position) to solve the balance problem.

### Weight Shift

**Fig. 2.**
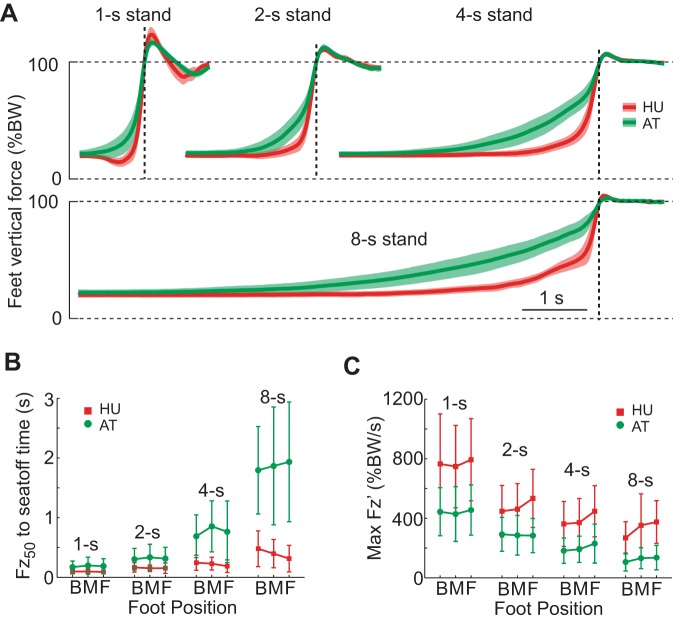
Weight shift. *A*: time course of F_*z*_ for chair rises from the middle foot position at each movement speed for healthy untrained (HU; red) and Alexander technique (AT; green). The shaded area indicates the 95% confidence interval (CI), and the dotted line indicates seat-off (SO). *B*: *T*_ws_ for all conditions (±SD). B, M, and F, back, middle and forward foot position, respectively. *C*: $F_{z}^{\prime }$ max for all conditions. While AT gradually increased F_*z*_ up through SO, HU rapidly and abruptly increased feet force just before SO, which was particularly pronounced for slow trials. BW, body weight.

For all movement conditions HU had a shorter duration, more rapid increase in feet force compared with AT (Fig. 2*A*). As shown in Fig. 2*B*, the F_*z*_ rise time, *T*_ws_, was relatively shorter in HU than AT across conditions [group: *F*(1,18) = 21.0, *P* < 0.001; see also Table 1]. This rise time was generally longer for slower instructed movement speeds [speed: *F*(1.23,22.1) = 54.6, *P* < 0.001]. However, HU did not prolong *T*_ws_ much for slower conditions (range 0.1–0.4 s) compared with AT (range 0.2–1.9 s) [group × speed: *F*(1.23,22.1) = 14.7, *P* < 0.001], which is consistent with this group's biphasic, gradual-then-abrupt feet-loading for slow movements.

**Table 1.** Statistical significance for weight-shift variables, CoM velocity, CoM-CoP distance, and maximal leg joint moments

	*T*_mov_	*T*_ws_	F_*z*_^′^ max	*V*_com_ Maximum	*X*_CoM-CoP_	Hip Moment Maximum	Knee Moment Maximum
Group	*F*(1,18) = 6.42	*F*(1,18) = 21.0	*F*(1,18) = 13.3	*F*(1,17) = 32.0	*F*(1,17) = 13.3	*F*(1,18) = 0.22	*F*(1,18) = 0.40
	*P* = 0.02	*P* < 0.001†	*P* = 0.002*	*P* < 0.001†	*P* = 0.002*	*P* = 0.65	*P* = 0.84
Speed	*F*(1.43,25.7) = 483.5	*F*(1.23,22.1) = 54.6	*F*(1.49,26.8) = 56.2	*F*(1.54,26.3) = 170.7	*F*(1.6,26.7) = 120.8	*F*(1.59,28.5) = 13.01	*F*(1.45,26.2) = 35.43
	*P* < 0.001†	*P* < 0.001†	*P* < 0.001†	*P* < 0.001†	*P* < 0.001†	*P* < 0.001†	*P* < 0.001†
Foot	*F*(2,36) = 3.82	*F*(2,36) = 0.56	*F*(1.68,30.1) = 11.4	*F*(1.4,23.8) = 54.5	*F*(1.34,22.8) = 35.4	*F*(1.5,26.6) = 102.4	*F*(1.45,26.04) = 5.29
	*P* = 0.03	*P* = 0.57	*P* < 0.001†	*P* < 0.001†	*P* < 0.001†	*P* < 0.001†	*P* = 0.019
Group × speed	*F*(1.43,25.7) = 2.68	*F*(1.23,22.1) = 27.6	*F*(1.49,26.7) = 2.18	*F*(1.54,26.3) = 0.93	*F*(1.57,26.7) = 0.54	*F*(1.59,28.54) = 4.50	*F*(1.45,26.2) = 1.17
	*P* = 0.10	*P* < 0.001†	*P* = 0.14	*P* = 0.39	*P* = 0.55	*P* = 0.03	*P* = 0.31
Group × foot	*F*(2,36) = 3.59	*F*(2,36) = 3.26	*F*(1.68,30.1) = 3.75	*F*(1.4,23.8) = 6.95	*F*(1.34,22.8) = 9.97	*F*(1.48,26.6) = 0.041	F(1.45,26.04) = 0.41
	*P* = 0.04	*P* = 0.052	*P* = 0.04	*P* = 0.008*	*P* = 0.002*	*P* = 0.92	*P* = 0.61
Speed × foot	*F*(2.7,49.7) = 0.48	*F*(2.7,50) = 0.33	*F*(4.31,77.5) = 2.11	*F*(6,102) = 1.21	*F*(4.98,84.6) = 0.53	*F*(4.03,72.45) = 3.55	*F*(4.61,82.88) = 3.30
	*P* = 0.68	*P* = 0.79	*P* = 0.083	*P* = 0.31	*P* = 0.75	*P* = 0.01*	*P* = 0.011*
Group × speed × foot	*F*(2.76,49.7) = 2.23	*F*(2.7,50) = 1.20	*F*(4.31,77.5) = 0.81	*F*(6,102) = 0.835	*F*(4.98,84.6) = 1.00	*F*(4.03,72.45) = 0.73	*F*(4.61,82.88) = 0.30
	*P* = 0.10	*P* = 0.32	*P* = 0.53	*P* = 0.55	*P* = 0.43	*P* = 0.58	*P* = 0.90

*T*_mov_, movement time; *T*_ws_, weight-shift rise time; F_*z*_^′^ max, maximal rate of combined-foot vertical loading; *V*_com_, CoM velocity; *X*_CoM-CoP_, center of mass (CoM) minus center of pressure (CoP).

* *P* < 0.01.

† *P* < 0.001.

The $F_{z}^{\;\prime }$ max, which occurred on average shortly before SO in both groups (HU: −0.08 ± 0.03 s; AT: −0.09 ± 0.06 s), was roughly twice that for HU than AT across conditions [Fig. 2*C*; group: *F*(1,18) = 13.3, *P* = 0.002]. In general, feet-loading rate slowed for slower instructed durations [speed: *F*(1.49,26.8) = 56.2, *P* < 0.001]; however, the large intergroup difference in $F_{z}^{\;\prime }$ max meant that HU had a higher maximum loading rate for 8-s trials (332.3 ± 161.4% BW/s) than AT did for 2 s (287.5 ± 116.7% BW/s). $F_{z}^{\;\prime }$ max was affected by foot placement, being greater for anterior foot positions when the CoM had to travel further forward [foot position: *F*(1.68,30.1) = 11.4, *P* < 0.001].

### CoM Motion

**Fig. 3.**
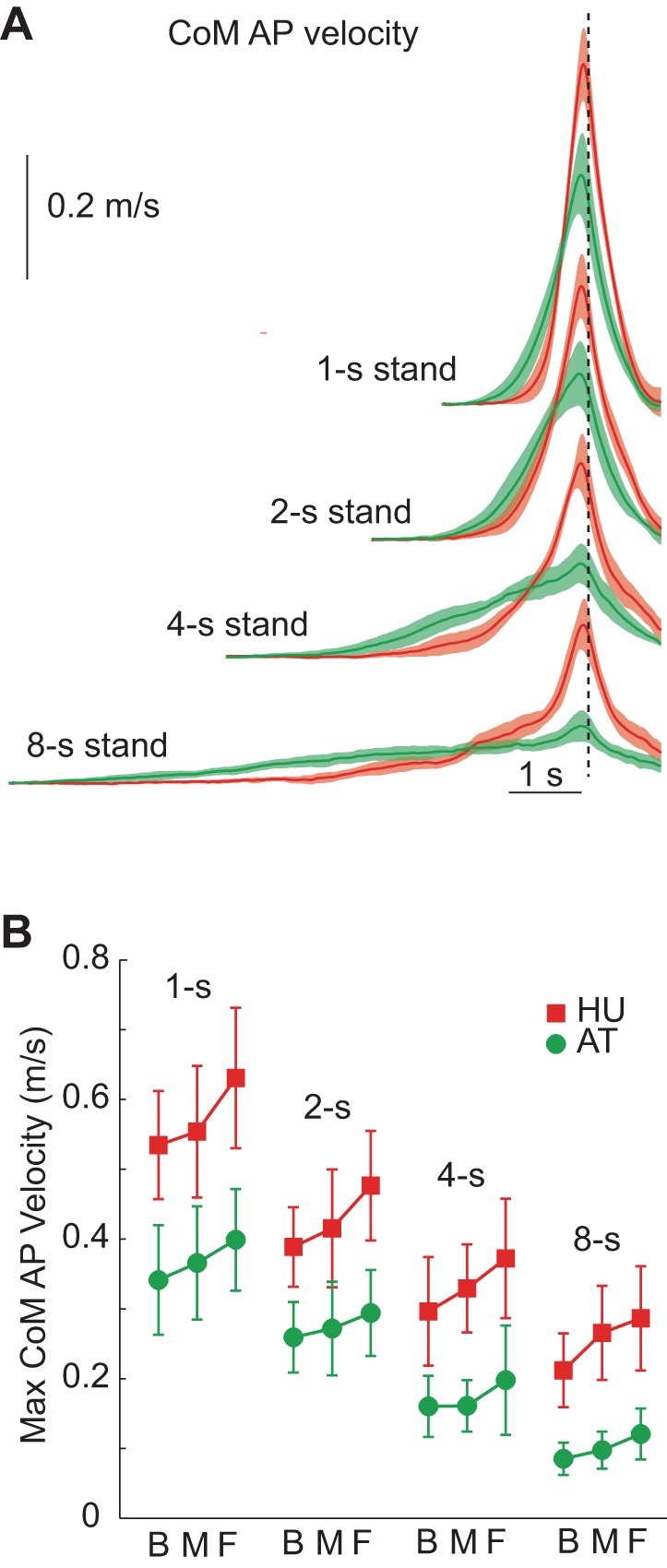
Center-of-mass (CoM) velocity (*V*_CoM_). *A*: time course of *V*_CoM_ for HU (red) and AT (green) while standing up from the middle foot position at each speed. The shaded area indicates 95% CI, and the dotted line indicates SO. *B*: maximal horizontal *V*_CoM_ across all movement conditions. HU had a greater maximal velocity than AT for all conditions. Note that for the slower conditions, HU had a low initial velocity but large increase just before SO, thereby violating the task instruction to stand up at a uniform speed. AP, anteroposterior.

For faster, 1- to 2-s chair rises, both groups displayed a marked increase in forward *V*_CoM_ just prior to SO (Fig. 3*A*). For the slowest movements, the AT group maintained an almost constant *V*_CoM_ from the start to SO, whereas HU seemed compelled to increase it abruptly just before SO. This indicates that, despite the experimenter's repeated emphasis, HU were not able to comply with the instruction to stand up at a uniform speed. Subjects were generally aware of and acknowledged this inability. In general, the maximal *V*_CoM_ closely preceded SO (by 0.11 ± 0.03 s for HU and 0.36 ± 24 s for AT) and also preceded $F_{z}^{\;\prime }$ max. However, HU had a much higher (∼2×) *V*_CoM_ than AT across all conditions [Fig. 3*B*; group: *F*(1,17) = 543.3, *P* < 0.001]. As expected, maximal *V*_CoM_ decreased for slower movements [speed: *F*(1.54,26.3) = 170.72, *P* < 0.001].

For both groups, the maximal *V*_CoM_ was larger for more anterior foot positions [foot position: *F*(1.4,23.8) = 54.5, *P* < 0.001], indicating that the speed increase prior to SO was related to the balance constraint. Foot position affected maximum *V*_CoM_ more for HU than AT [group × foot position: *F*(1.4,23.8) = 6.95, *P* = 0.008], suggesting that the latter group was better able to resolve the balance constraint (see below) without resorting to the use of exaggerated forward momentum of the body.

### Achieving Bipedal Balance

**Fig. 4.**
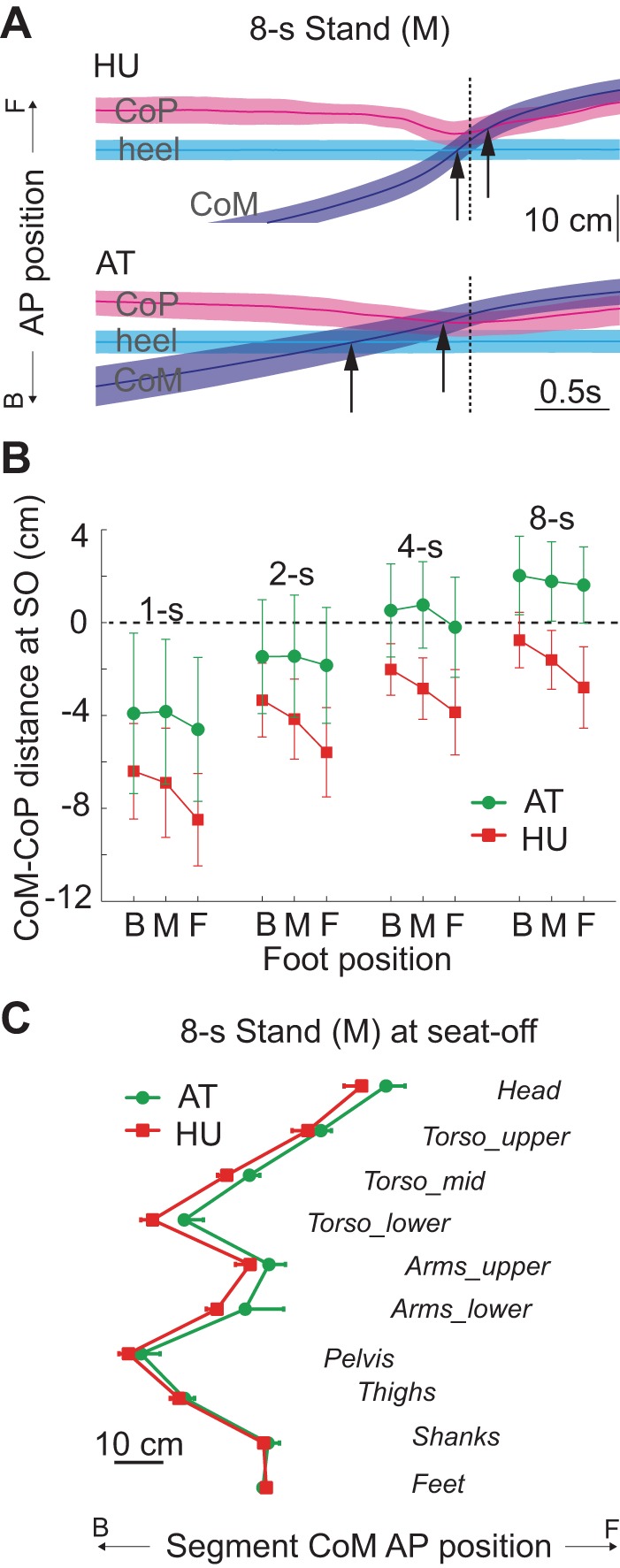
Bipedal balance. *A*: trajectories of horizontal CoM (blue), combined-feet CoP (pink) and heel position (cyan) for the 8-s middle (M) foot condition for both groups. Shaded areas indicate 95% CI. Black arrows demarcate when the CoM reaches the heel and CoP, achieving forward balance. Note that at SO (dotted line) the CoM was anterior to the heel and CoP for AT, but only the heel for HU. *B*: CoM-CoP distance at SO (±SD). Positive values indicate the CoM was anterior to the CoP. Note that AT achieved a more forward CoM position than HU by SO for all conditions. Static stability at SO (i.e., CoM-Co*P* > 0) only occurred for the AT group (4- and 8-s conditions). *C*: AP positions of the segmental mass centroids at SO, relative to CoP, for the 8-s middle condition. The vertical axis is arranged to approximate vertical segmental position. The arms were placed just above the pelvis to avoid overlapping with the torso. AT's anterior whole body CoM at SO was largely due to more anterior upper body segments.

The difference in the way the two groups solved the balance problem is illustrated in Fig. 4, which shows the relationship between the CoM, CoP and heel positions. To maintain balance and avoid fall-back, either the CoM position must be forward of the CoP at SO, or the CoM must have sufficient velocity such that the body's forward momentum will take the CoM over the feet a short time later (Pai and Patton 1997). Consistent with this, both groups had relatively more positive *X*_CoM-CoP_ values (more anterior CoM with respect to CoP at SO) for slower movement speeds [Fig. 4*B*; speed: *F*(1.57,26.7) = 120.8; *P* < 0.001]. Across all conditions, however, AT teachers' *X*_CoM-CoP_ was more positive by several centimeters [group: *F*(1,17) = 13.3; *P* = 0.002], and this forward CoM position is consistent with their lower forward velocity. Notably, for 4- and 8-s conditions, AT had positive values of *X*_CoM-CoP_ (CoM anterior to CoP at SO), so this group did not require any forward velocity to maintain balance. In contrast, HU's CoM was behind the CoP at SO for all conditions, thus requiring forward momentum to achieve dynamic stability over the feet.

As expected, positioning the feet anteriorly made *X*_CoM-CoP_ less positive, as the CoM had further to travel to reach the feet [foot position: *F*(1.34,22.8) = 35.4; *P* < 0.001]. However, AT were less affected by foot placement than HU [group × foot position: *F*(1.34,22.8) = 9.97; *P* = 0.002], indicating they were more capable of moving the CoM forward throughout weight shift than HU. In general, AT's forward CoM can be attributed to upper body segments (Fig. 4*C*), which is consistent with greater hip joint flexion at SO.

### Maximal Leg Extensor Moments

**Fig. 5.**
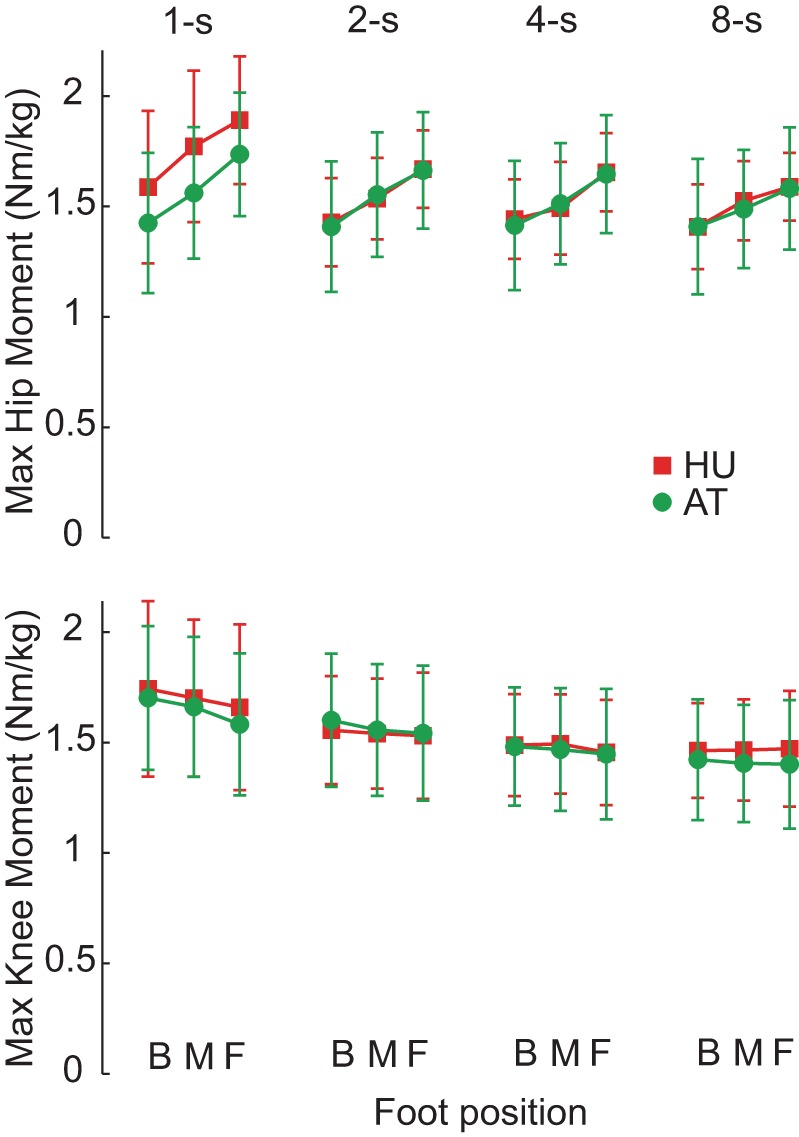
Maximal hip and knee moments during STS. Similar values occurred for HU (red) and AT (green), indicating AT's smoother coordination did not require increased strength. Moreover, that lower maximal moments occurred for slower, more difficult conditions suggests that strength did not underlie difficulty standing up slowly and smoothly.

From these results, it appears that the HU group was unable to achieve the slow continuous movements displayed by the AT group. One reason for this could be that the AT performance, particularly for the slow movements that were the most challenging, required greater strength. However, as shown in Fig. 5, joint moments were lower for slower speeds [speed: hip, *F*(1.59,28.54) = 13.01, *P* < 0.001; knee, *F*(1.45,26.15) = 35.43, *P* < 0.001], suggesting that slower movements required less rather than more strength. Moreover, there were no significant group differences or interactions involving group, suggesting that AT performance did not require greater strength overall.

### Relation Between Balance, *V*_CoM_ and F_*z*_ Loading Rate

**Fig. 6.**
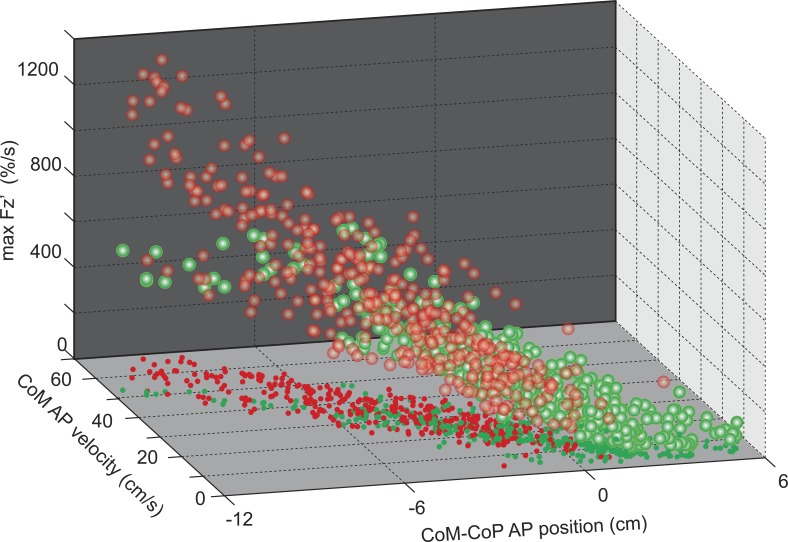
Interrelation between weight shift, *V*_CoM_ and bipedal balance. Each subject's condition averages for $F_{z}^{\prime }$ max, *V*_CoM_ at SO and CoM-center-of-pressure (CoP) distance at SO. Red dots with white center indicate data from HU, and green dots with white center indicate AT. Smaller solid dots mark the projection of data onto the horizontal plane. For each group, data for the slowest conditions are toward the front lower right and fastest conditions are in the rear upper left. The majority of variance across condition and group (91.5%) is described by a single principle component revealing a strong relationship between these three variables. However, AT data are shifted along this component toward a further forward CoM, slower *V*_CoM_ and lower $F_{z}^{\prime }$ max relative to HU.

A strong interrelationship was present between $F_{z}^{\prime }$ max, *V*_CoM_ at SO and *X*_CoM-CoP_ (Fig. 6). Principal component analysis yielded that a single component (0.975 × *V*_CoM_ at SO, 0.944 × $F_{z}^{\;\prime }$ max, −0.952 × *X*_CoM-CoP_) accounted for 91.5% of the variance in these variables. The pairwise correlations were given by *r* = 0.886 between *V*_CoM_ and $F_{z}^{\;\prime }$ max, −0.909 between *V*_CoM_ and *X*_CoM-CoP_, and −0.823 between $F_{z}^{\;\prime }$ max and *X*_CoM-CoP_. All pairwise correlations were significant at *P* < 0.001 level. This strong relationship suggests a single unitary phenomenon underlies the variation in these variables across speeds, foot position and groups.

## DISCUSSION

### Difficulty Standing Up Slowly and Smoothly

The results show that young HU adults have more difficulty than the cohort of AT teachers when attempting to stand up smoothly from a seated position. HU began by slowly leaning the upper body forward, but just before SO they abruptly sped up followed by a rapid loading of their feet. In contrast, AT showed a gradual, prolonged weight shift to the feet and with only relatively small increases in velocity and feet force around SO. Similar features were observed in a previous study on AT rising at a self-selected speed (Cacciatore et al. 2011b). HU were aware of their jerky, discontinuous movement and commented on their inability to stand up smoothly for slow rises. AT were more capable, but still they reported it challenging to rise smoothly for the slowest movements with the most forward feet position. It is important to emphasize that HUs in the present study understood the task requirements and were consistently encouraged to move smoothly, but in general were unable to comply. Furthermore, the contrast between groups was most prominent for the unnaturally slow movements that took up to 8 s to execute. This argues against the jerkiness of HU being due either to a misunderstanding of task instructions to stand up smoothly or to habitual movement patterns. The inability to rise smoothly was not related to strength differences or to differences in force distribution between joints. The maximal leg extensor moments were nearly identical between groups, and the slower, more challenging, conditions had the lowest hip and knee extensor moments (see also Pai and Rogers 1990; Yoshioka et al. 2009). This suggests the limitation was in the central nervous system's control of the action.

This difficulty rising slowly and smoothly has not been reported previously for HU, presumably because prior studies did not investigate chair rises from feet forward positions that were sufficiently slow. For some studies the “slow” condition did not exceed 2 s (Doorenbosch et al. 1994; Gross et al. 1998; Mourey et al. 2000). For others, the instruction to stand up “as slowly as possible” resulted in movement durations of only 2.5 s (Pai and Rogers 1990) or 3–4 s (Kotake et al. 1993). One report did consider very slow movements of up to 7 s (Yoshioka et al. 2007) by artificially slowing the kinematics of faster rises (<2 s) using computer simulation. However, this method assumes STS coordination is invariant with movement speed, which is not supported by our data.

### Constraints of Balance and Posture

The two quantities we used for assessing jerkiness of performance were forward *V*_CoM_ and rate of change of vertical feet force ${\mathrm{(F}}_{z}^{\prime }$ max). They were correlated with each other as well as with the horizontal distance between the CoM and the position of the CoP under the feet at SO. The relationship was such that the further back the CoM was relative to the CoP, the higher the *V*_CoM_ and $F_{z}^{\prime }$ max. While both groups showed a similar relationship, HU were shifted toward backwards mass positions, higher CoM velocities and faster weight shift.

Part of this relationship can be explained by the constraint of balance, which requires that the body's CoM ultimately reaches a position directly above the feet. If the CoM is behind the feet (or more strictly behind the CoP) at the point of SO, then balance can only be achieved by the body having sufficient forward momentum to propel it over the feet dynamically (Mourey et al. 2000; Pai and Patton 1997). The further back the body is at SO, the greater its forward velocity must be. From this explanation it seems that HU's higher *V*_CoM_ could simply have been due to their more posterior CoM position at SO. However, this does not explain why they did not come further forward and satisfy the task instructions. Their posterior position did not seem to be due to structural restrictions, as they did not approach normative maximal range of motion [HU hip flexion at SO = 98.9 ± 14.8°, 8-s feet forward condition; typical adult range of motion = ∼120°, (e.g., Roach and Miles 1991)].

The other part of the relationship can be explained by the constraint of posture, which requires that the body does not collapse under its own weight. The legs must take the body's weight as it lifts from the chair, requiring forceful leg extension moments to prevent collapse. However, the need for extension moments conflicts with the need to flex the leg joints to bring the CoM over the feet to balance. HU appeared to solve the conflict by delaying the extension moments as long as possible so as not to interfere with generation of forward momentum. This left little time for F_*z*_ to reach BW before the body left the seat, thus causing a high $F_{\mathrm{z.}}^{\prime }$ However, this does not explain why HU were unable to continue flexing their legs while simultaneously generating extensor moments in the same way as AT.

### The Role of Stiffness

Can differences in postural stiffness between the groups (Cacciatore et al. 2011a) provide a single explanation both for why HU were unable to bring their CoM far enough forward and why HU activated extensor muscles later in the action, and thereby explain HU's jerkiness when rising from a chair? To answer this, we constructed an eight-segment sagittal-plane neuromechanical model of the body to simulate STS. Upper body moments were passively generated by springs and dampers. Leg joints had both active and passive components. Importantly, activity-dependent stiffness was implemented for the hip and knee so that each joint's stiffness was proportional to its active torque. Thus hip and knee stiffness increased during weight shift as these extensor moments increased (Granata et al. 2002; Weiss et al. 1988; Zhang et al. 1998). Variation in co-contraction was simulated by changing the coefficient between torque and stiffness.

First, the model was made to stand up successfully from a seated position using physiologically realistic parameter values and anthropometrics (see appendix for model details). Driving the hip and knee active torque profiles with a ramp-shaped time course of activation caused the model to rise in a reasonable fashion. Next, the activity-dependent stiffness of individual joints was varied to examine its effect. The model's success in rising was observed by the CoM-CoP trajectories, as movement failures occurred due to the vertical projection of the whole body CoM not reaching the CoP position. This caused the body to fall backwards as the legs were extended.

**Fig. 7.**
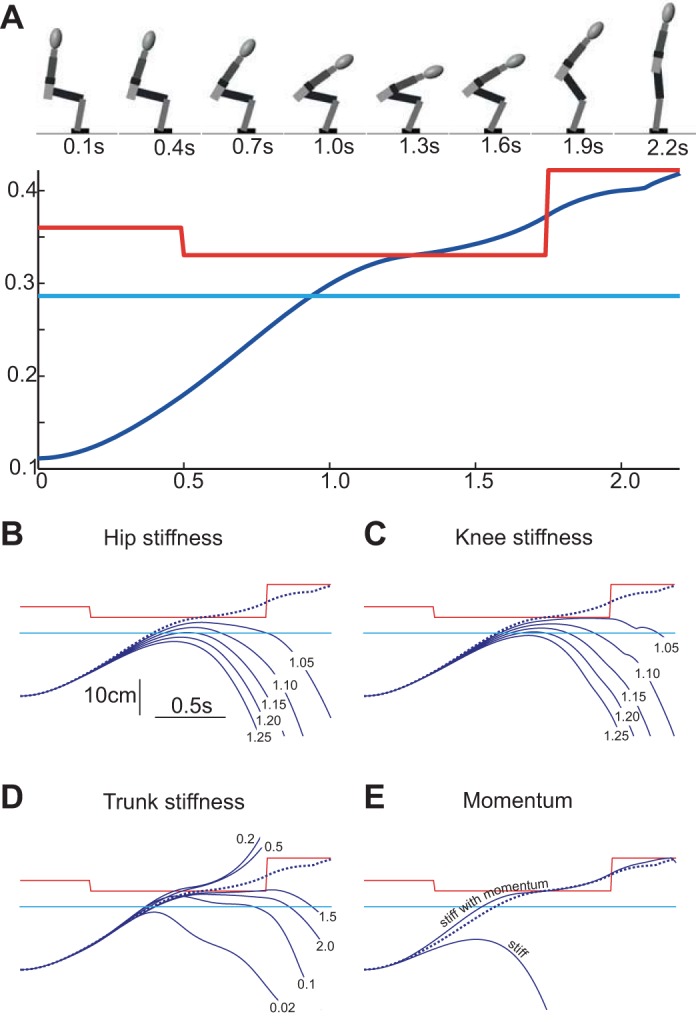
Effect of stiffness on model CoM trajectory. *A*: successful chair-rise simulation. The figurine shows the model geometry at 0.3-s intervals. The CoM (blue trace) moved anteriorly, crossing the heel (cyan trace) and under-foot CoP (red trace). This forward motion was driven by gravity acting to incline the upper body, which also flexed the hip, knee and ankle (the latter two joints flexed from the forward translation of the femur as the pelvis tilted). Active hip and knee extensor torques weighted the feet and extended the legs. Active ankle torque moved the CoP backwards then forwards to approximate that measured experimentally. Note that CoM motion was not due to active hip flexion. Active hip flexor torque was not observed experimentally for 2- to 8-s durations, so it was not included in the model. *B*: the effect of hip stiffness on CoM motion. Dashed line shows the CoM trajectory for the “default” simulation in *A*. Solid blue lines show the result of simulations where hip stiffness was increased by a factor of 1.05–1.25. All increases caused “sit-back” failures as the CoM failed to reach the CoP. Note that, because the hip stiffness was activity dependent, the restriction is analogous to difficulty stretching active hip extensors. *C*: the effect of increased knee stiffness (1.05–1.25× default) on CoM trajectory. Knee stiffness restricted forward body motion by reducing thigh, shank and pelvis motion. As in the previous panel, this restriction represents difficulty driving eccentric contractions of the legs during weight shift. *D*: the effect of trunk stiffness on CoM motion. The passive stiffness of all trunk joints was scaled by between 0.02 and 2× the default value. Both high and low trunk stiffness acted to hinder CoM motion. This complex dependency likely resulted from altered force transmission and trunk kinematics. *E*: the effect of momentum on stiff model behavior. A “stiff” model was created by increasing hip (activity-dependent stiffness 1.5× default), knee (activity-dependent stiffness 1.25× default) and trunk stiffness (passive value 2× default). This caused a prominent sit-back failure as the CoM failed to reach the CoP. By delaying weight shift (i.e., activating hip and knee torques 0.09 and 0.13 s later, respectively), the same stiff model could be made to successfully rise. The delay increased forward trunk momentum, which overcame the higher leg stiffness during weight shift and caused the CoM to reach the CoP. As the minimal delays that enabled forward balance were used, the “stiff with momentum” trace represents the lower bound for weight-shift delay and *V*_CoM_ (slope) for the stiff model to successfully stand. Note that the disparity between stiff and default model joint stiffness (e.g., 1.5×) underestimated that measured between HU and AT (Cacciatore et al. 2011a).

The results from a typical set of simulations are shown in Fig. 7. The model stood up successfully with realistic values of activity-dependent stiffness for the hip and knee joints (Fig. 7*A*). However, the movement failed with relatively small increases in this stiffness of only 5% for either the hip joint (Fig. 7*B*) or the knee joint (Fig. 7*C*). This demonstrates that increases in active stiffness of the leg joints can act to prevent the CoM from travelling sufficiently far forward, thus causing a failure to satisfy balance requirements.

The stiffness of the trunk may also play a role in the STS action. It was notable that AT displayed very low spinal bending around SO compared with HU, a phenomenon that has been observed previously (Cacciatore et al. 2011b; Johnson et al. 2010; Tully et al. 2005). We simulated differences in trunk stiffness in our model by changing an overall scaling factor, without altering the relative stiffness of the three trunk and neck “joints.” As shown in Fig. 7*D*, change in passive trunk stiffness did indeed influence the STS action, but the results were not simple. The action could be made to fail by decreasing the trunk stiffness as well as by increasing it. This aspect of the model requires further investigation, possibly by incorporating active elements and varying the relative stiffness along the trunk. Nonetheless, it serves our present purpose of illustrating that trunk stiffness contributes to the overall performance.

The final step was to investigate whether these stiffness-related failures of the model could be remedied by delaying the onset of weight shift. To test this, the active hip and knee stiffness and passive trunk stiffness were all increased together, using values that on their own caused a STS failure (Fig. 7*E*). Not surprisingly, these stiffness values caused the model to fail when using the activation times for Fig. 7*A*. However, delaying the onset of hip and knee extensor activation profiles led to a higher *V*_CoM_ and allowed the stiff model to stand successfully. Thus a need to compensate for greater stiffness, notably, 80% greater in HU than AT for the hip (Cacciatore et al. 2011a), can explain HU's delay in weight shift and reliance on higher velocity.

### Implications for Neural Control of Posture

The reason why stiffness greatly affected the model behavior stems from the need to continue moving the body mass forward as the feet are weighted by leg extensor moments. For this to happen, positive work has to be performed on the active hip and knee extensor muscles to stretch them as they generate force. This can come from upper body momentum or from gravitational forces acting on the upper body. In the absence of substantial upper body momentum, as required by very slow and smooth rises, gravity becomes the major player. However, there are two requirements for trunk gravitational forces to overcome the active leg extensor activity to flex the hip and knee joints. First, to transmit gravitational force across the trunk to the hip joint, the trunk must be sufficiently stiff to prevent it from yielding under the gravitational bending moment, which increases with trunk incline. Second, the stiffness of leg joints must be sufficiently low for the gravitational torque to exceed the torque produced by the resistive forces in active leg extensor muscles. Note that this resistive torque is not necessarily the same as the net extensor torque of the joint. For example, co-contraction of antagonist muscles can increase the mechanical resistance to stretch of extensors without altering the net extensor torque.

We, therefore, suggest that neural processes that affect stiffness are critical to performance of STS. But what neural processes affect stiffness? Differences in joint stiffness affecting STS could simply result from the patterns of co-contraction that are programmed together with all the other muscle activity required to stand up from a seated position, i.e., the phasic motor plan. However, if such a phasic control process were the source of stiffness, one might expect subjects to readily modify their motor plan and STS performance through practice. Yet HU appeared unable to alter their behavior across numerous experimental trials. An alternative hypothesis is that stiffness results from a separate neural process that coexists and interacts with the process issuing phasic bursts of muscle activity. This is an idea that is more in keeping with the observations that AT and HU exhibit different patterns of stiffness when simply standing without phasic movements (Cacciatore et al. 2011a). It is also consistent with the observation that HU tends to use higher levels of postural stiffness than necessary (Di Giulio et al. 2013). Such stiffness could be caused by processes that directly regulate joint stiffness (Kearney et al. 1997; Ludvig et al. 2007) or joint configuration (Di Giulio et al. 2013), which are distinct from movement control (Burdet et al. 2001; Franklin et al. 2003). Moreover, these processes are both involved with the ongoing, tonic maintenance of body posture in the face of external forces, which we refer to as postural tone.

How might postural tone interact with movement and account for differences in STS coordination between HU and AT? Throughout the STS movement, postural tone must support the mass of the upper body and prevent it from collapsing against gravity. As the axial musculature is highly complex and redundant, this postural support can be achieved in different ways, with differing spatial distributions (Claus et al. 2009; O'Sullivan et al. 2006) and different dynamic control (Cacciatore et al. 2011a; Gurfinkel et al. 2006). The specific way this support is achieved creates a stiffness distribution across the body or “postural frame.” This may not be localized to the portion of the body being supported, but could extend to more distal regions through intersegmental interactions (Caronni and Cavallari 2009; Franzen et al. 2011; Gurfinkel et al. 1995). Thus, HU's difficulty could be due to their heightened leg stiffness caused by poor postural control. Alternatively or additionally, poor postural control could lead to a slack spine with insufficient stiffness to transfer gravitational force to stretch leg extensors. On the other hand, it is possible that AT's facility for smooth, near steady-state movement coordination is due to their ability to dynamically modulate the postural frame. The results of this study, that stiffness observed in a postural context can account for movement difficulty, suggest that for healthy adults the specific way antigravity support is regulated can act to interfere with the overall coordination of an everyday, functional movement. In the case of STS, resistance within a subcomponent task, moving the CoM forward, can plausibly affect the whole movement by altering the ability to satisfy a global balance constraint.

### Implications for Movement Coordination

Poor postural regulation has the capacity to affect movement profoundly. Here we have presented evidence from rising from a chair, but other movements have similar mechanical conflicts. For example, stair climbing requires flexing leg joints to move the CoM over the foot while generating large extensor moments to rise up. Eccentric contractions are necessary for many everyday movements, such as gait, squatting, lunging, sitting down in a chair, etc. This potential interference may be exacerbated in the elderly. For example, when rising from a chair, the elderly have a CoM position that is even more posterior at SO (Mourey et al. 2000), which can cause “sit-back” failures and lead to functional disability (Riley et al. 1997). While their difficulty has been attributed to fear of falling (Mourey et al. 2000) or low strength (Bernardi et al. 2004; Schenkman et al. 1996), it may stem from a poor ability to drive eccentric contractions due to excessive stiffness. If this is the case, perhaps training programs should not address strength or teach greater momentum (Schlicht et al. 2001; Schot et al. 2003), but instead address postural control to reduce its interference with movement, leading to more efficient coordination.

## GRANTS

This study was supported by Medical Research Council (G0802073) and Wellcome Trust (084870/Z/08/Z).

## DISCLOSURES

No conflicts of interest, financial or otherwise, are declared by the author(s).

## AUTHOR CONTRIBUTIONS

Author contributions: T.W.C., O.S.M., A.P., and B.L.D. conception and design of research; T.W.C. and A.P. performed experiments; T.W.C., O.S.M., and B.L.D. analyzed data; T.W.C., O.S.M., and B.L.D. interpreted results of experiments; T.W.C. and B.L.D. prepared figures; T.W.C. and B.L.D. drafted manuscript; T.W.C., O.S.M., A.P., and B.L.D. edited and revised manuscript; T.W.C., O.S.M., A.P., and B.L.D. approved final version of manuscript.

## Supplementary Material

Video S1

Video S2

## Appendix

### Model Description

The neuro-mechanical forward model was constructed in SimMechanics and Simulink R2013b (The Mathworks, Natick, MA) using second-generation block elements. A sagittal-plane model of the body was constructed from eight segments that were all rectangular, except for an elliptical head. Segmental dimensions and masses are given in Table A1 and reflected the anthropometrics used in Visual 3D for a typical subject (methods).

**Table A1.** Inertial properties of model segments

	Length, cm	Thickness, cm	Mass, kg
Head/neck	26	15	4.8
Top trunk	10	8	9.8
Middle trunk	25	10	14.8
Bottom trunk	8	12	12.3
Pelvis	14	11	8.4
Thigh	43	10	11.8
Shank	40	8	7.6
Foot	22	6	1.7

Intersegmental joints were implemented in the model by revolute (pin) joints that connected the mid-edges of adjacent segments. The thigh segment was connected to the pelvis with a vertical offset of 11 cm so the hip joint was above the chair in the initial seated position. Chair height and foot position were adjusted so the simulation started with the model body configuration in the experimental middle foot position. Chair-pelvis contact forces were generated in Simscape. The foot segment was fixed rigidly to the floor, and ankle torque was constrained to ensure the CoP remained within the foot boundary.

Upper body joint moments were generated passively by springs and dampers. Stiffness values for the default condition (Fig. 7*A*) were 5, 10, 15 and 25 Nm/° for the neck, upper back, mid-back and low-back joints, respectively. The corresponding viscosities were 0.1, 0.2, 0.3 and 0.5 Nm·s/°. Lower body moments consisted of both passive and active components. Hip, knee and ankle joints had a fixed viscosity of 0.05 Nm/°. Hip and knee joints had a stiffness that varied their active joint torque. For the knee, the default value (i.e., Fig. 7*A*) was set at 0.05°^−1^ based on Granata et al. (2002) and Zhang et al. (1998). We were unable to find published experimental values for the hip, which was set at 0.02°^−1^. Ankle joint stiffness was not included in the model, as there were no eccentric ankle contractions in either HU or AT before SO, and, therefore, ankle stiffness would not impede forward CoM motion before lift-off.

The simulation began with the upper body inclined several degrees so that gravity acted to lean the trunk forward and drive leg joint flexion. Because the hip joint was located above the point of chair contact (due to its 11-cm vertical offset), pelvis inclination caused the femur to translate forward and induce both knee and ankle joint flexion. Simple delayed ramp-shaped activation profiles were sufficient to make the model rise successfully from the chair. For the default condition, the active hip torque was given by the following: delay = 0.12 s, slope = 35.5 Nm/s and saturation = 90 Nm. Likewise, the active knee torque was given by the following: delay = 0.07 s, slope = 25 Nm/s and saturation = 40 Nm. While such simple torque profiles were not able to precisely match the time course of increasing hip and knee gravitational torques, and thus achieve very slow 8-s chair rises, they were sufficient to explore the effect of joint stiffness on coordination. Ankle torque was controlled to approximate that measured experimentally in both AT and HU: a flexor torque of 20 Nm between 0.5 and 1.75 s, followed by an extensor torque of 42 Nm.
